# Fucoxanthin from microalgae *Phaeodactylum tricornutum* inhibits pro-inflammatory cytokines by regulating both NF-κB and NLRP3 inflammasome activation

**DOI:** 10.1038/s41598-020-80748-6

**Published:** 2021-01-12

**Authors:** A-Hyeon Lee, Hye-Yoon Shin, Jong-Hwi Park, Song Yi Koo, Sang Min Kim, Seung-Hoon Yang

**Affiliations:** 1 Department of Medical Biotechnology, College of Life Science and Biotechnology, Dongguk University, Seoul, 04620 Republic of Korea; 2 School of Life Science, Handong University, Pohang, Gyeongbuk 37554 Republic of Korea; 3 KIST Gangneung Institute of Natural Products, Gangneung, 210-340 Republic of Korea; 4 Department of Food and Nutrition, and Research Institute of Human Ecology, Seoul National University, Seoul, 08826 Republic of Korea; 5 Division of Bio-Medical Science and Technology, KIST School, Korea University of Science and Technology, Seoul, 02792 Republic of Korea

**Keywords:** Neuroscience, Neurology

## Abstract

Pro-inflammatory cytokines such as IL-1β, IL-6, and TNF-α are mediated by the activation of various kinds of signaling pathways in the innate immune system. Particularly, NF-κB and NLRP3 inflammasome signaling are involved in the production and secretion of these cytokines. Each signaling is participated in the two steps necessary for IL-1β, a representative pro-inflammatory cytokine, to be processed into a form secreted by cells. In the priming step stimulated by LPS, pro-IL-1β is synthesized through NF-κB activation. Pro-IL-1β cleavages into mature IL-1β by formed NLRP3 inflammasome in the activation step induced by ATP. The mature form of IL-1β is subsequently secreted out of the cell, causing inflammation. Moreover, IL-6 and TNF-α are known to increase in NLRP3 inflammasome-mediated conditions. Here, we found that fucoxanthin, one of the major components of *Phaeodactylum tricornutum*, has an inhibitory effect on NF-κB and NLRP3 inflammasome activation induced by the combination of LPS and ATP in bone marrow-derived immune cells as well as astrocytes. Fucoxanthin, which is abundant in the EtOH fraction of *Phaeodactylum tricornutum* extracts, has shown to have less cell toxicity and found to decrease the production of major pro-inflammatory cytokines such as IL-1β, IL-6, and TNF-α. Fucoxanthin has also shown to suppress the expression of cleaved caspase-1 and the oligomerization of ASC, which are the main components of the NLRP3 inflammasome. Furthermore, phosphorylated IκBα and pro-IL-1β expression decreased in the presence of fucoxanthin, suggesting that fucoxanthin can negatively regulate the priming step of inflammasome signaling. Thus, our results provide reliable evidence that fucoxanthin may serve as a key candidate in the development of potential therapeutic agents for inflammatory diseases as well as neurodegenerative diseases caused by NF-κB and NLRP3 inflammasome activation.

## Introduction

Inflammation is a mechanism of the body to protect against adverse substances, such as invading pathogens, damaged cells, or environmental irritants^1^. The function of inflammation is to localize and destroy harmful agents, and to get rid of infected or damaged tissue components, allowing the body to begin reparation^2^. The acute inflammatory response is short-lived and normally beneficial against potential threats with minimal cellular damage, whereas a long-standing chronic inflammatory response can lead to tissue damage and ultimately its destruction via sustained accumulation of pro-inflammatory mediators, such as cytokines and chemokines^3^. This type of prolonged inflammation can contribute to the development of diseases such as: rheumatoid arthritis, type 2 diabetes, and neurodegenerative diseases (Alzheimer’s disease and Parkinson’s disease)^4,5^.

Inflammasomes are cytosolic molecular components of the innate immune system which, when activated, triggers the inflammatory response. IL-1β is the primary pro-inflammatory cytokine produced by inflammasome activation, particularly by the NLRP3 (NOD-, LRR-, and pyrin domain-containing protein 3) inflammasome. IL-1β is first synthesized as a pro-form by stimulation of pathogen-associated molecular patterns (PAMPs) such as LPS on specific pattern-recognition receptors (PRRs) of immune cells through the activation of NF-κB signaling (priming step). Pro-form IL-1β is then matured through the NLRP3 inflammasome activation, additionally stimulated by damaged-associated molecular patterns (DAMPs) such as ATP or uric acid (activating step)^6,7^. During NLRP3 inflammasome activation, adaptor protein ASC (apoptosis-associated speck-like protein containing caspase recruitment domain or CARD) oligomerizes to form a complex that is referred to as the ‘speck’. Oligomerized ASC recruits in pro-caspase-1, one of the components of the NLRP3 inflammasome, into the complex and converts it into active caspase-1 by proteolytic cleavage. Activated caspase-1 subsequently cleaves pro-IL-1β into mature IL-1β^8^.

IL-6 and TNF-α are representative pro-inflammatory cytokines as well as IL-1β and have diverse immune functions that regulate inflammatory responses in various kinds of disease models. IL-6 is known for downstream target of IL-1β, consequently activated and increased in patients with NLRP3 inflammasome related diseases^9^. Recently, it has been reported that IL-6 and NLRP3 inflammasome are main regulator in immune response stimulated by pathogen and viral infection such as SARS-CoV-2^10^. Additionally, TNF-α also has a function on the activation of NLRP3 inflammasome components in inflammatory diseases. In the study of TNF-α deficient dendritic cell, the transcriptional expression of pro-caspase-1 and pro-IL-1β are significantly reduced. Therefore, IL-6 and TNF-α are important mediators for NLRP3 inflammasome activation^11^.

The activation of inflammasomes are often indirectly evaluated through the detection and analyses of inflammasome components and, or pro-inflammatory cytokines in various types of immune cells such as bone marrow-derived macrophages (BMDMs) and bone marrow-derived dendritic cells (BMDCs) and CNS-resident immune cell. Particularly astrocytes, which are the most abundant immune cell type in the brain, have been identified as active components in neuroinflammation; furthermore, evidence that sustained inflammation in astrocytes is associated with neurodegenerative diseases have been found^12,13^.

Fucoxanthin (Fx), one of the major carotenoids present in microalgae, is known to have a variety of biological properties, such as anti-oxidant, anti-cancer, anti-obesity, and anti-inflammation^14,15^. Recent studies suggest that fucoxanthin is a useful therapeutic agent for inflammatory diseases because it relieves inflammation by inhibiting NF-κB activation in vitro and *in vivo*^16,17^. Industrial production of fucoxanthin has been mainly through macroalgae such as *Laminaria japonica* and *Undaria pinnatifida*. However, due to various existing problems^18,19^, microalgae *Phaeodactylum tricornutum* has recently been proposed as a major source for fucoxanthin production^20,21^.

In this study, we investigated the effects of fucoxanthin extracted from *Phaeodactylum tricornutum* on LPS/ATP-stimulated inflammation using multiple cell models, which included bone marrow-derived macrophages and dendritic cells as well as astrocytes. The cytotoxicity of crude extract, fractions separated by different organic solvents, and fucoxanthin was initially confirmed. Furthermore, the level of pro-inflammatory cytokines such as IL-1β, IL-6, and TNF-α was measured by ELISA to determine the effects of fucoxanthin. Additionally, the expression of inflammatory genes and molecular mechanisms were analyzed to examine whether fucoxanthin reduces IL-1β secretion by inhibiting NF-κB and NLRP3 inflammasome activation, thus allowing further understanding of the physiological functions of fucoxanthin in relation to inflammatory diseases and neurodegenerative diseases.

## Results

### Microalgae *Phaeodactylum tricornutum* extracts containing fucoxanthin lower IL-1β secretion in bone marrow-derived immune cells

**Figure 1:**
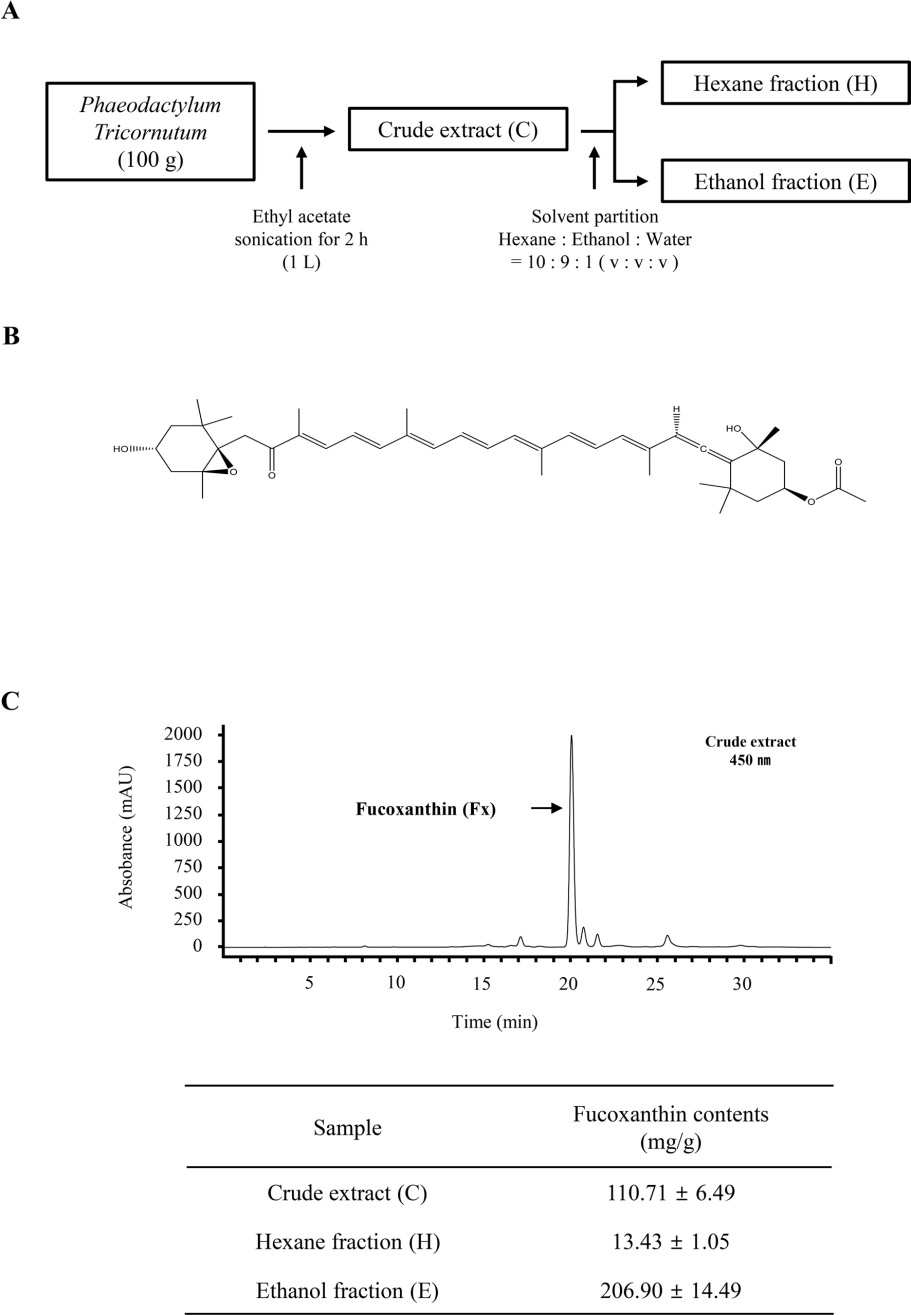
Extraction process and chemical structure of fucoxanthin from microalgae *Phaeodactylum tricornutum* (**A**,**B**). Quantification of fucoxanthin contents in three fractions of *Phaeodactylum tricornutum* extracts (**C**). C, Crude; H, Hexane; E, Ethanol.

**Figure 2:**
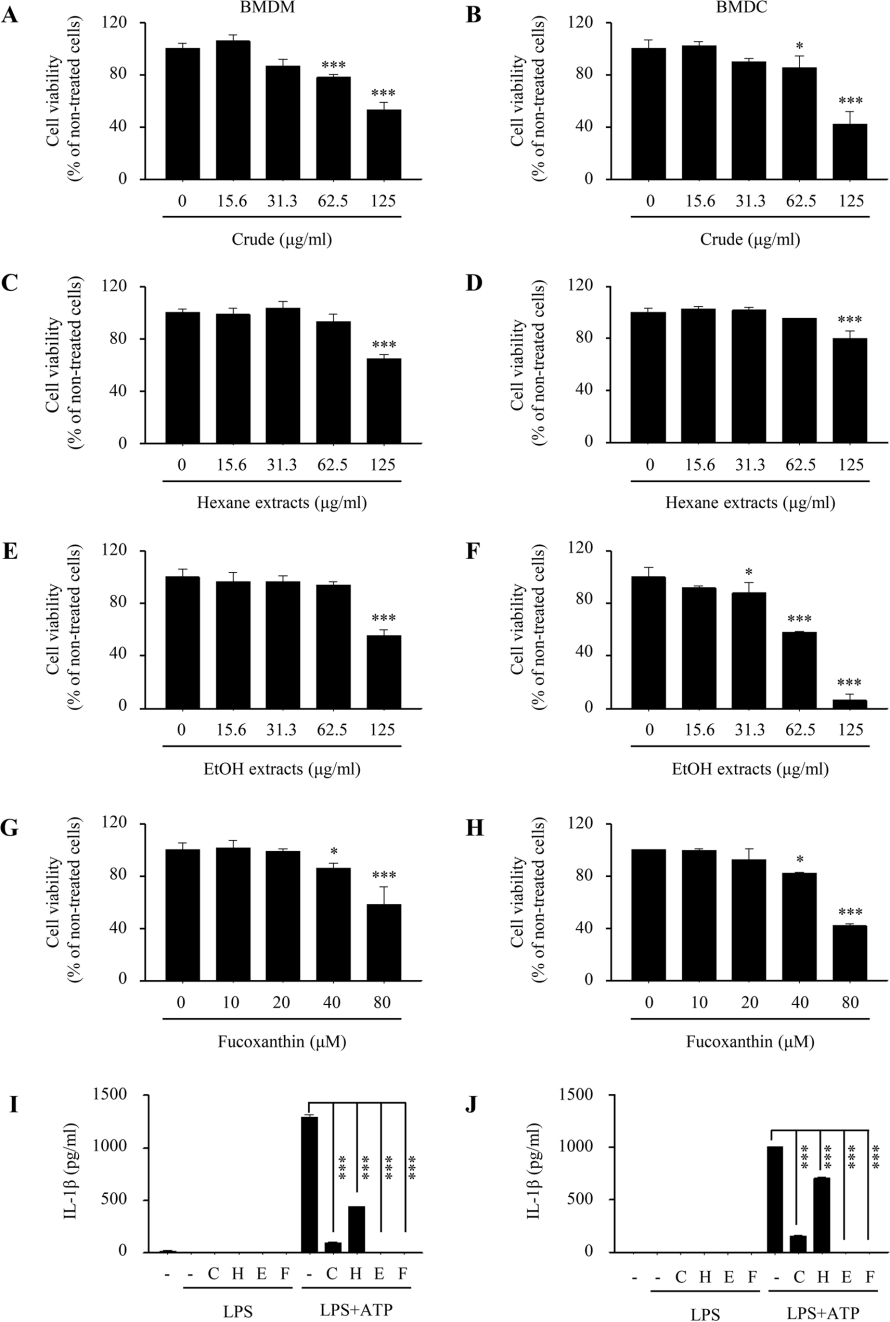
Effect of *Phaeodactylum tricornutum* extracts on cell viability and IL-1β production in bone marrow-derived macrophages (BMDMs) and dendritic cells (BMDCs). (**A–H**) BMDMs and BMDCs were treated with crude (**A**,**B**), hexane (**C**,**D**), ethanol (**E**,**F**) extracts and fucoxanthin (**G**,**H**) at various concentrations. Cell viability was then measured by MTT assay. (**I**,**J**) BMDMs and BMDCs were pre-treated with crude (62.5 μg/ml), hexane (62.5 μg/ml), ethanol (31.3 μg/ml), and fucoxanthin (40 μM) 4 h prior to treatment of LPS (1 μg/ml) and ATP (5 mM). C, Crude; H, Hexane; E, Ethanol. Data are presented as mean ± SEM. *P ≤ 0.05 and ***P ≤ 0.001 (one-way ANOVA followed by Bonferroni's post hoc comparisons tests).

The crude extract (C), hexane (H) fractions, and ethanol (E) fractions containing different amounts of fucoxanthin were produced from *Phaeodactylum tricornutum* biomass as shown in Fig. 1A,B. Quantification results in Fig. 1C showed that fucoxanthin contents in these samples were the highest in ethanol fraction (20.6% w/w) and the lowest in hexane fraction (1.3%, w/w). These extracts were then assessed for cell viability using the MTT assay. Each extract was shown to have dose-dependent cytotoxicity but less toxic effect at concentrations below 62.5 μg/ml (Fig. 2A–F). Fucoxanthin, found most abundantly in the EtOH extracts, also has no cytotoxicity at concentrations below 40 μM (Fig. 2G,H). To evaluate the biological and physiological effects of *Phaeodactylum tricornutum* extracts on immune cells, we pre-treated each extract to BMDMs and BMDCs for 4 h before LPS and ATP stimulation to induce inflammation. Then the level of IL-1β, a major pro-inflammatory cytokine induced by LPS and ATP, was measured by ELISA. We found that pre-treatment with each extract reduced IL-1β production in both BMDMs (Fig. 2I) and BMDCs (Fig. 2J). Especially, fucoxanthin and EtOH extracts with high fucoxanthin concentration had a dramatic effect on reducing the amount of IL-1β compared to other extracts, suggesting that fucoxanthin can effectively inhibit IL-1β secretion on bone marrow-derived immune cells.

### Fucoxanthin significantly reduces the gene expression related to inflammation and the production of proinflammatory cytokines

**Figure 3:**
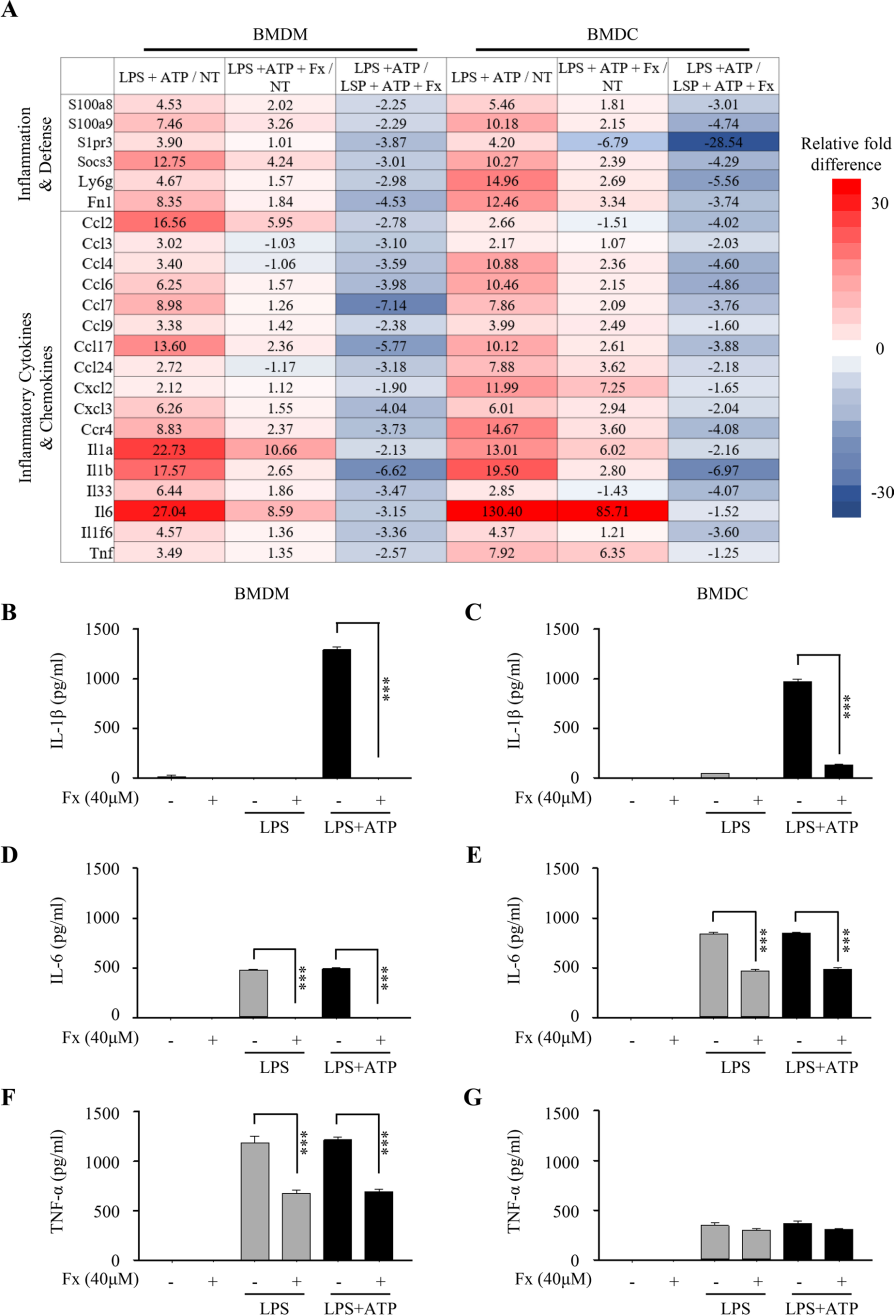
Gene expression profiling analysis and pro-inflammatory cytokine measurement in LPS or LPS/ATP-treated bone marrow-derived macrophages (BMDMs) and dendritic cells (BMDCs). (**A**) Functional groups of genes expression in BMDMs and BMDCs treated with LPS and ATP in the presence or absence of fucoxanthin. Red and blue color indicates expression levels higher or lower than non-treated cells, respectively. NT, non-treated cells. (**B–G**) BMDMs and BMDCs were pre-treated with fucoxanthin (40 μM) 4 h prior to treatment of LPS (1 μg/ml) and ATP (5 mM). The secretion of the IL-1β (**B**,**C**), IL-6 (**D**,**E**) and TNF-α (**F**,**G**) by BMDMs and BMDCs was measured by ELISA. Data are presented as mean ± SEM. ***P ≤ 0.001 (one-way ANOVA followed by Bonferroni's post hoc comparisons tests).

Inflammatory response by LPS and ATP stimulation is accompanied by the up-regulation of various inflammatory mediators such as cytokines and chemokines as well as IL-1β^22^. To examine the change in gene transcript expression level of inflammatory mediators in immune cells by fucoxanthin treatment, we applied Affymetrix Whole transcript Expression arrays. We found that some of the inflammation-related genes, including inflammatory cytokines, increased in expression by LPS and ATP application in both BMDMs and BMDCs compared to non-treated cells, whereas the expression of those genes was significantly reduced in cells treated with fucoxanthin before LPS and ATP stimulation (Fig. 3A). Moreover, we aimed to determine whether fucoxanthin can not only affect gene expression but also the resulting secretion of inflammatory mediators such as pro-inflammatory cytokines (IL-1β, IL-6, and TNF-α), which were measured by ELISA assay. In BMDMs, fucoxanthin decreased LPS/ATP-induced IL-1β, IL-6, and TNF-α secretion (Fig. 3B,D,F). Similarly, in BMDCs the level of those cytokines decreased with treatment of fucoxanthin with the exception of TNF-α (Fig. 3C,E,G). These findings suggest that fucoxanthin can successfully inhibit not only IL-1β but also other pro-inflammatory cytokines such as IL-6 and TNF-α in both gene and protein expression levels.

### Fucoxanthin negatively blocks the LPS/ATP-induced NLRP3 inflammasome activation

**Figure 4:**
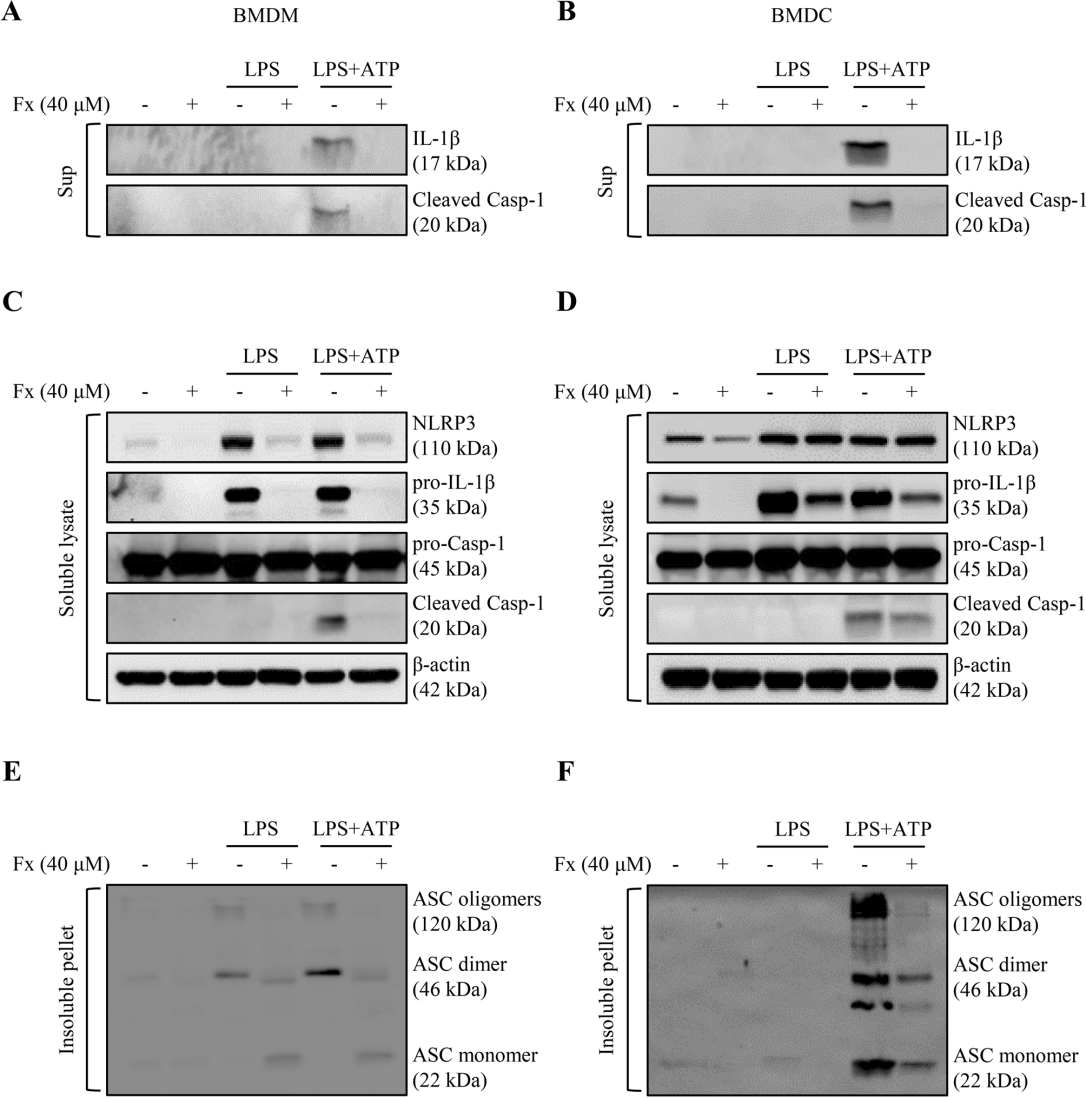
Western blot analysis of NLRP3 inflammasome activation in bone marrow-derived macrophages (BMDMs) and dendritic cells (BMDCs). Both types of cells were treated with LPS (1 μg/ml) and ATP (5 μM), and the changes with or without fucoxanthin (40 μM) pre-treatment 4 h ago were compared. (**A**,**B**) Expression levels of the mature form of IL-1β and proteolytically processed caspase-1 in the culture media of BMDMs (**A**) and BMDCs (**B**). (**C**,**D**) Western blot analysis of NLRP3 inflammasome component expression in LPS/ATP treated BMDMs (**C**) and BMDCs (**D**) with or without fucoxanthin. (**E**,**F**) ASC oligomerization in cross-linked pellet of BMDMs (**E**) and BMDCs (**F**) was also analyzed by immunoblot.

The release of IL-1β is associated with a series of activation of the NLRP3 inflammasome and caspase-1^7^. To investigate whether fucoxanthin effects NLRP3 inflammasome activation, we performed a western blot analysis of BMDMs and BMDCs supernatant. We found that fucoxanthin effectually restrained mature IL-1β and cleaved caspase-1 production by LPS/ATP stimulation (Fig. 4A,B). We then explored the signaling mechanism by which fucoxanthin blocks NLRP3 inflammasome activation where the expression level of NLRP3 inflammasome components such as NLRP3, ASC, and caspase-1 were measured in both BMDMs and BMDCs by western blot. As a result, a significant reduction of NLRP3, pro-IL-1β, and cleaved caspase-1 was observed in BMDMs by the treatment of fucoxanthin, whereas BMDC exhibited no significant fucoxanthin-induced inhibitory effect only in NLRP3 (Fig. 4C,D).

As the activation of NLRP3 promotes ASC oligomerization to form the insoluble speck, the detection of ASC oligomerization is one of the frequent indicators for inflammasome activation^8^. To examine whether fucoxanthin prevents ASC oligomerization, BMDMs and BMDCs treated by LPS and ATP were lysed in NP-40 lysis buffer and the pellets were chemically cross-linked, followed by immunoblots detecting ASC. ASC oligomers were detected, and results showed that the pellets of fucoxanthin treated cells contained significantly lower amounts of ASC dimers and oligomers compared with the LPS and ATP treated cells (Fig. 4E,F). Taken together, these findings suggest that fucoxanthin can play an immunomodulatory role by regulating IL-1β processing through inhibition of NLRP3 inflammasome assembly.

### NF-κB signaling, the priming step of inflammasome activation, is suppressed by fucoxanthin

**Figure 5:**
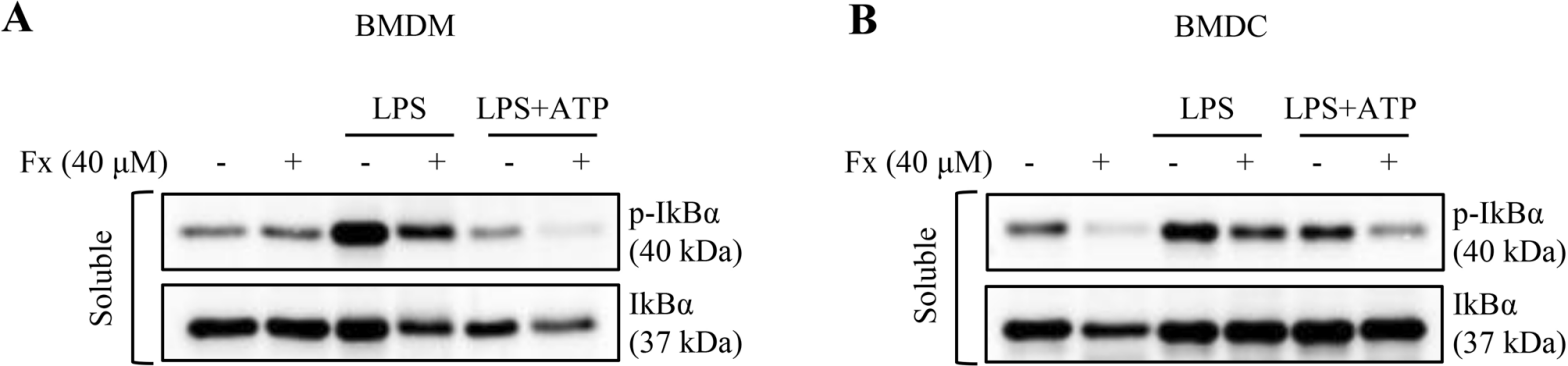
Regulation of NF-κB signaling by fucoxanthin. (**A**,**B**) Cellular expression levels of phosphorylated IκBα expression in bone marrow-derived macrophages (BMDMs) (**A**) and dendritic cells (BMDCs) (**B**) treated with LPS alone or a combination of LPS and ATP with or without fucoxanthin.

NF-κB mediates the priming signal of the NLRP3 inflammasome, which induces transcriptional expression of pro-IL-1β in response to LPS. Thus, inducible degradation of IκBα, an inhibitor of NF-κB, through site-specific phosphorylation by the canonical pathway causes NF-κB to trigger pro-IL-1β expression^23^. To directly assess the effect of fucoxanthin on NF-κB activation involved in the priming step of the NLRP3 inflammasome, we examined the phosphorylation of IκBα in both LPS and LPS/ATP-stimulated BMDMs and BMDCs. The level of phosphorylated IκBα increased by LPS or LPS/ATP stimulation appeared to return to normal levels when treated with fucoxanthin in both BMDMs and BMDCs (Fig. 5A,B). Therefore, this suggests that fucoxanthin can regulate the priming step of the NLRP3 inflammasome by inhibition of IκBα phosphorylation.

### Secretion of pro-inflammatory cytokines was also inhibited by the treatment of fucoxanthin in astrocytes

**Figure 6:**
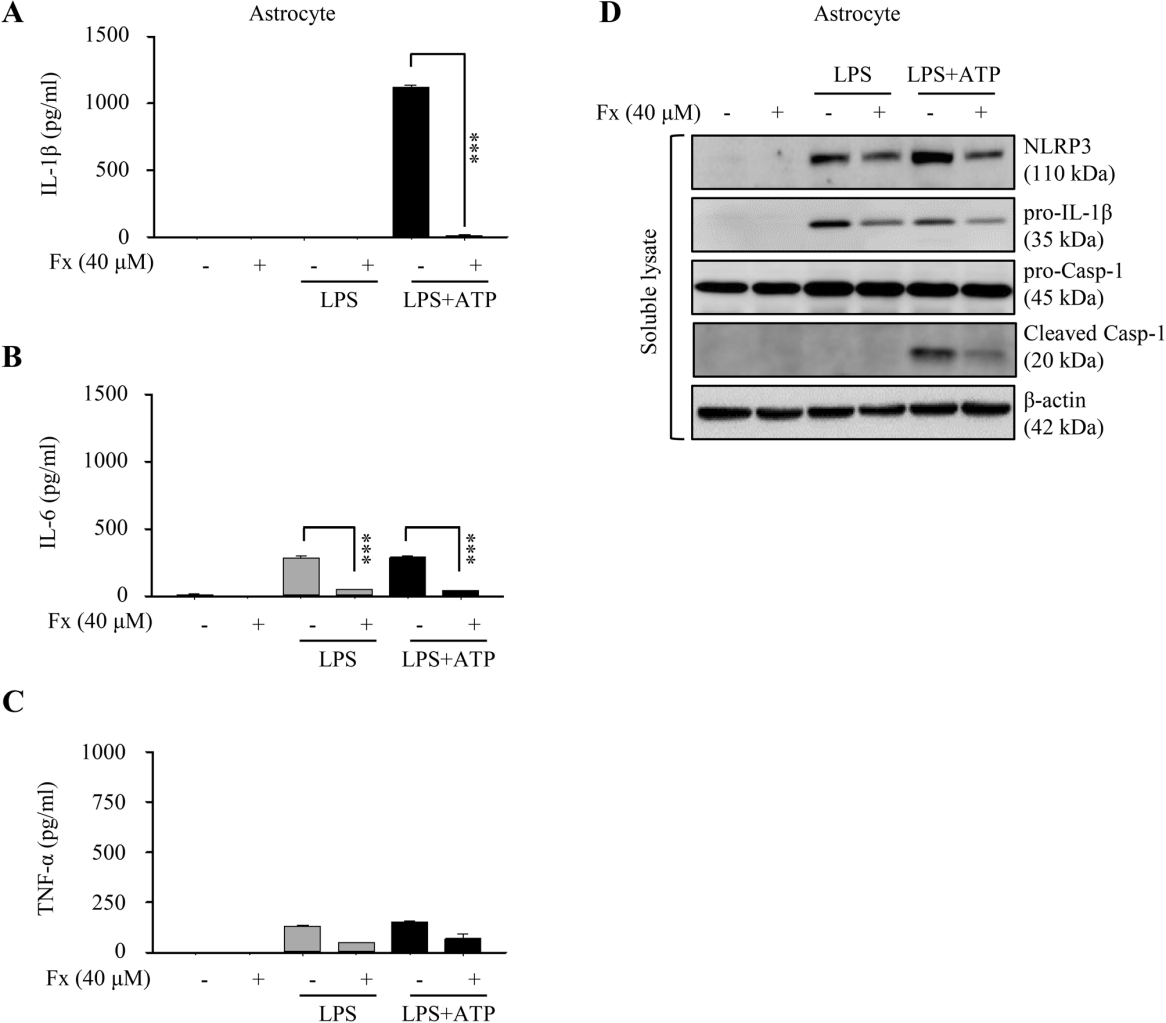
Cytokine production and NLRP3 inflammasome activation in astrocytes. (**A-C**) Measurement of cytokine concentration for IL-1β (**A**), IL-6 (**B**), and TNF-α (**C**) in astrocytes treated with LPS and ATP in the presence or absence of fucoxanthin. (**D**) Protein expression analysis of NLRP3 inflammasome components in astrocytes when stimulated with LPS or LPS/ATP after fucoxanthin treatment.

Astrocytes also have been reported to have pro-inflammatory potential and play crucial roles in regulating neuroinflammation with implications for neurodegenerative diseases, such as the Alzheimer’s disease. Furthermore, inflammatory cytokine activity has been observed in astrocytes, similar to bone marrow-derived immune cells^24^. To assess the effects of fucoxanthin on neuroinflammation, we measured alteration in the level of pro-inflammatory cytokines by ELISA. We found that the quantity of IL-1β and IL-6 secreted from LPS/ATP-induced astrocytes had been significantly reduced by fucoxanthin, except for TNF-α, which was slightly decreased by fucoxanthin treatment (Fig. 6A–C). Consistent with the above results, decreased expression of NLRP3, pro-IL-1β, and cleaved caspase-1 for each stimulus caused by fucoxanthin was also detected (Fig. 6D). Collectively, these findings suggested that fucoxanthin effectively inhibits pro-inflammatory cytokines release and NLRP3 inflammasome activation in neural cells as well as immune cells, showing the potential for application as a treatment for neuroinflammatory diseases.

## Discussion

Here, we report that fucoxanthin extracted from *Phaeodactylum tricornutum* inhibits the pro-inflammatory cytokines (IL-1β, IL-6, and TNF-α) secretion of BMDMs and BMDCs in response to LPS and ATP while not showing cytotoxicity. Multiple gene transcripts related to immune defense and inflammatory cytokines induced by LPS and ATP were also down-regulated by fucoxanthin treatment in BMDMs and BMDCs. Furthermore, the mechanism of fucoxanthin’s effects on the NLRP3 inflammasome activation was investigated, and it was discovered that the protein expression of NLRP3, ASC, and cleaved caspase-1 had been decreased. Moreover, the level of phosphorylated IκBα was restored through fucoxanthin treatment in LPS/ATP-induced BMDMs and BMDCs, indicating that fucoxanthin can regulate NF-κB signaling, which is the priming step for inflammasome activation. Similar results were shown in the astrocytes, demonstrating the inhibitory effect of fucoxanthin in neuroinflammation.

Inflammation, generally considered to be a beneficial immunological response to pathogens, is also closely associated with the progress of multiple diseases when prolonged. Therefore, understanding the mechanism of inflammation is critical for the treatment of inflammatory disease. NF-κB has a complex role in various cell types and is implicated in the pathogenesis of many inflammatory diseases. Rheumatoid arthritis (RA), associated with chronic inflammation, is mainly mediated by NF-κB activation in synovial tissue^25^. The involvement of NF-κB signaling in inflammatory bowel disease (IBD), multiple sclerosis (MS), Atherosclerosis has been demonstrated in several studies^25–27^. The NLRP3 inflammasome is also known to play a vital role in inflammation activation and has been implicated in various inflammatory diseases including neurodegenerative diseases. Thus, the inhibition of NF-κB and NLRP3 inflammasome has become a promising target for the treatment of inflammatory disease^28,29^. So far, several NLRP3 inhibitors have been developed but none have been clinically utilized, and the curative effects of these inhibitors have not been sufficiently confirmed^6,28,29^.

Furthermore, the majority of the current inhibitors being developed are synthetic compounds that have a potential risk of side-effects^30^. However, the safety of fucoxanthin derived from macroalgae or microalgae was confirmed by many clinical researches in the animals^30^ and even in the human^31^. In addition, many methods of fucoxanthin production was suggested, using macroalgae^32^ and microalgae^33^ for appropriate commercial application. Therefore, fucoxanthin, a marine carotenoid, can be a potential resource for the treatment of inflammatory disease-related with NF-κB and NLRP3 inflammasome. Currently, there are only a few studies examining the therapeutic application of fucoxanthin. There are even fewer of those that focus on the anti-inflammatory properties of fucoxanthin, as most studies are concentrated on the anti-obese and anti-oxidant effects of fucoxanthin^34,35^. Studies on the inhibitory effects of fucoxanthin on the NLRP3 inflammasome are particularly scarce. Although one recent study reported that the treatment of fucoxanthin combined with rosmarinic acid, affected the activation of NLRP3 inflammasome^36^, our study on the inhibitory effects of fucoxanthin was conducted in depth as a singular compound with particular emphasis on the signaling mechanism of the NLRP3 inflammasome. Therefore, our study suggests that fucoxanthin from *Phaeodactylum tricornutum* microalgae may be a potential singular therapeutic agent for inflammatory diseases as well as neurodegenerative diseases caused by NLRP3 inflammasome activation.

Additionally, though our results are promising, further investigation is required to examine whether fucoxanthin would also be beneficial in animal models with inflammatory disease and neuroinflammation related neurodegenerative diseases. As an example, due to recent study developments that shows a correlation of NLRP3 inflammasome activation in APP/PS1 transgenic mice, which is one of Alzheimer’s disease (AD) animal models^37^, it is possible to predict that fucoxanthin may be used to improve the pathological symptoms observed in the AD mice model, such as memory impairment. Thus, the expansion on the potential effect of fucoxanthin in the treatment of inflammatory and neurodegenerative diseases related to NF-κB signaling and NLRP3 inflammasome, is encouraged for further studies.

## Materials and methods

### Animals

Wilde-type (male, C57BL/6, 6–8-week-old) mice were obtained from the Jackson Laboratory (Bar Harbor, Maine, USA). All mice were housed in a laboratory animal breeding room at Dongguk University. All animal experiments were approved by the Institutional Animal Care and Use Committee at Dongguk University and performed in the regulation of institutional guidelines.

### Chemicals and reagents

Antibodies used for western blot were IL-1β (AF-401-NA, R&D System), Caspase-1 (AG-20B-0042-C100, AdipoGen Life Science), NLRP3 (AG-20B-0014-C100, AdipoGen Life Science), ASC (AG-25B-0006-C100, AdipoGen Life Science), phospho-IκBα (Ser32) (#2859, Cell Signaling), IκBα (sc-371, Santa Cruz) and β-actin (#3700, Cell Signaling).

### Preparation of extracts from *Phaeodactylum tricornutum*

The freeze-dried sample of *Phaeodactylum tricornutum* was provided from Algaetech (Gangneung, Korea). For extraction and partition, an analysis grade solvent purchased from Daejeong Inc. (Seoul, Korea) was utilized. All solvents for HPLC analysis were purchased Fisher Scientific (Springfield, NJ). The standard compound of all-*trans*-fucoxanthin was purchased from Sigma-Aldrich (St. Louis, MO, USA), and used for the control and the construction of calibration curve. During extraction, the freeze-dried powder of *Phaeodactylum tricornutum* (100 g) was obtained with ethyl acetate (1 L) through sonication for 2 h at room temperature. Solids were removed using a Whatman No.1 filter paper (Thermofisher, New Zealand) and the crude extract (C) was obtained by using a rotary evaporator at 35 °C. The crude extract was partitioned with *n*-hexane (200 mL) and 90% ethanol (200 ml) in a separation funnel. The upper and lower phase were evaporated at 35 °C to obtain hexane fraction (H) and ethanol fraction (E) as shown in Fig. 1A.

### Quantification of fucoxanthin

The fucoxanthin content was analyzed by HPLC using the Agilent 1260 series HPLC system. YMC carotenoid column (250 × 4.6 mm i.d. with 5-μm particle size; Waters, Ireland) was utilized for the separation. Methanol and water solvent system was employed for the mobile phase at a flow rate of 0.7 ml/min with a column temperature of 35 °C. The solvent gradient program was as follows: methanol/water ratio was increased from 90:10 to 100:0 over 20 min, and then 100% methanol was held for the next 5 min. The chromatogram was obtained at 450 nm and a standard compound of all-*trans*-fucoxanthin was used to construct a calibration curve in a range from 1 to 200 μg/ml for quantitative analysis as previously described^21^.

### Preparation of bone marrow-derived macrophages (BMDMs) and dendritic cells (BMDCs)

For the primary culture of BMDMs, the femur was isolated from 6–8-week-old mice. Femoral bone marrow cells were cultured at a density of 5 × 10^6^ cells with DMEM medium (Gibco) supplemented with 20% fetal bovine serum (Gibco), 30% L929 cell-conditioned medium, 50 μM 2-mercaptoethanol (Daejung), 100 units/ml penicillin, and 100 μg/ml streptomycin (Gibco) in a 100 mm bacteria dish. After a full day, nonadherent cells were collected and resuspended in a fresh medium. Aliquots of 1 × 10^6^ cells were cultured for 10 days at 37 °C and 5% CO_2_ in a 100 mm cell culture dish. Fresh culture medium was added on day 4 and replaced on day 7^38^.

In BMDCs culture, femoral bone marrow cells were cultured with RPMI 1640 medium (Gibco) supplemented with 10% fetal bovine serum (Gibco), 1 mM sodium pyruvate (Gibco), 1% nonessential amino acid solution (Gibco), 50 μM 2-mercaptoethanol (Daejung), 20 ng/ml GM-CSF (Peprotech), 100 units/ml penicillin, and 100 μg/ml streptomycin (Gibco) at a 100 mm bacteria dish (5 × 10^6^ cells) for 8 days. Fresh culture medium was added and replaced on day3 and day 6 respectively^39^.

### Preparation of astrocytes

The cerebral cortex and hippocampus of newborn C57BL/6 mice between day 0 and day 3 were dissected for primary astrocytes. These were minced in DMEM medium (Gibco) supplemented with 10% horse serum (Gibco), 10% fetal bovine serum (Gibco), 100 units/ml penicillin, and 100 μg/ml streptomycin (Gibco). After that, it was gently pipetted for single-cell suspension and transferred to a 60 mm cell culture dish pre-coated with poly-D-lysine (PDL) (Sigma) for 15 min. On the 3rd day of culture, cells were washed with PBS (Gibco) and supplied with fresh medium. It was replaced with fresh media again on day 6^40^.

### Cell viability assays

BMDMs (1 × 10^4^ cells/100 μl/well) and BMDCs (1 × 10^4^ cells/100 μl/well) were seeded into a 96-well plate to evaluate the cytotoxicity of crude, hexane, ethanol extract, and fucoxanthin by MTT assay from Promega. After treatment of each extract, 15 μl of dye solution was added and incubated for 4 h. 100 μl of Solubilization solution was then added and kept at room temperature for 12 h. The insoluble formazan was measured at 570 nm wavelength.

### Cytokine assays

Pro-inflammatory cytokine concentrations in BMDMs (2 × 10^6^ cells/2 ml/well), BMDCs (2 × 10^6^ cells/2 ml/well), and astrocytes (2 × 10^6^ cells/2 ml/well) seeded in 6-well plate were quantitated with IL-1β, TNF-α, and IL-6 ELISA kits from R&D Systems following the manufacturers’ instructions. Briefly, cells were pre-treated with 40 μM fucoxanthin for 4 h and stimulated with 1 μg/ml LPS (Invivogen) alone or a combination of l μg/ml LPS and 5 mM ATP (Invivogen).

### Western blot analysis

BMDMs, BMDCs, and astrocytes were lysed using lysis buffer containing 150 mM NaCl (Daejung), 50 mM Tris–HCl (pH 7.5) (Bio-Rad), 1% NP-40 (Sigma), 1 mM EDTA (Thermo), and protease inhibitor cocktail (PIC) (Roche). Each lysate was centrifuged at 6000 rpm for 15 min at 4 °C. The supernatants were used as soluble fractions and the pellets as insoluble fractions. The soluble fractions were directly dissolved in SDS containing sample buffer (Invitrogen) and applied to western blot analysis. The insoluble fractions were washed in cold PBS and crosslinked with 2 mM disuccinimidyl suberate (DSS) (Thermo) for ASC oligomerization. The cross-linked pellets were then centrifuged at 13,000 rpm for 15 min and were eluted by boiling with SDS containing sample buffer. Each fraction was separated by SDS–polyacrylamide gel electrophoresis. These were then transferred to a polyvinylidene difluoride membrane and detected by the indicated antibodies.

### Profiling of gene transcript expression

The Affymetrix Whole transcript Expression array was used to access gene expression in both BMDCs and BMDMs according to the manufacturer's protocol (GeneChip Whole Transcript PLUS reagent Kit). Briefly, RNA was extracted by using RNeasy Mini Kit (QIAGEN) and was applied to synthesize cDNA using the GeneChip WT (Whole Transcript) Amplification kit. Utilizing the GeneChip WT Terminal labeling kit, the cDNA was then labeled with biotin and TdT (terminal deoxynucleotidyl transferase). Labeled DNA was hybridized to the Affymetrix GeneChip Mouse 2.0 ST Array and stained on a GeneChip Fluidics Station 450, then scanned on a GCS3000 Scanner (Affymetrix).

## Supplementary Information

Supplementary Information.
