# Supplementary material for: Fucoxanthin from microalgae Phaeodactylum tricornutum inhibits pro-inflammatory cytokines by regulating both NF-κB and NLRP3 inflammasome activation
Source: Sci Rep. 2021 Jan 12;11:543. doi: 10.1038/s41598-020-80748-6 (PMC7803995; doi:10.1038/s41598-020-80748-6)
Supplement: Supplementary file 1 — Supplementary Information. [file 41598_2020_80748_MOESM1_ESM.docx]

**Supplementary Information**

**Fucoxanthin from microalgae *Phaeodactylum tricornutum* inhibits pro-inflammatory cytokines by regulating both NF-κB and NLRP3 inflammasome activation**

A-Hyeon Lee, Hye-Yoon Shin, Jong-Hwi Park, Song Yi Koo, Sang Min Kim, and Seung-Hoon Yang

* Corresponding Author

Seung-Hoon Yang, Department of Medical Biotechnology, College of Life Science and Biotechnology, Dongguk University, Seoul 04620, Republic of Korea
Tel.: +82 31 961 5156; fax: +82 31 961 5108.
E-mail address: shyang@dongguk.edu (S.H. Yang).

Supplementary Information:

Supplementary Figure 1

Supplementary Figure 2

Supplementary Figure 3


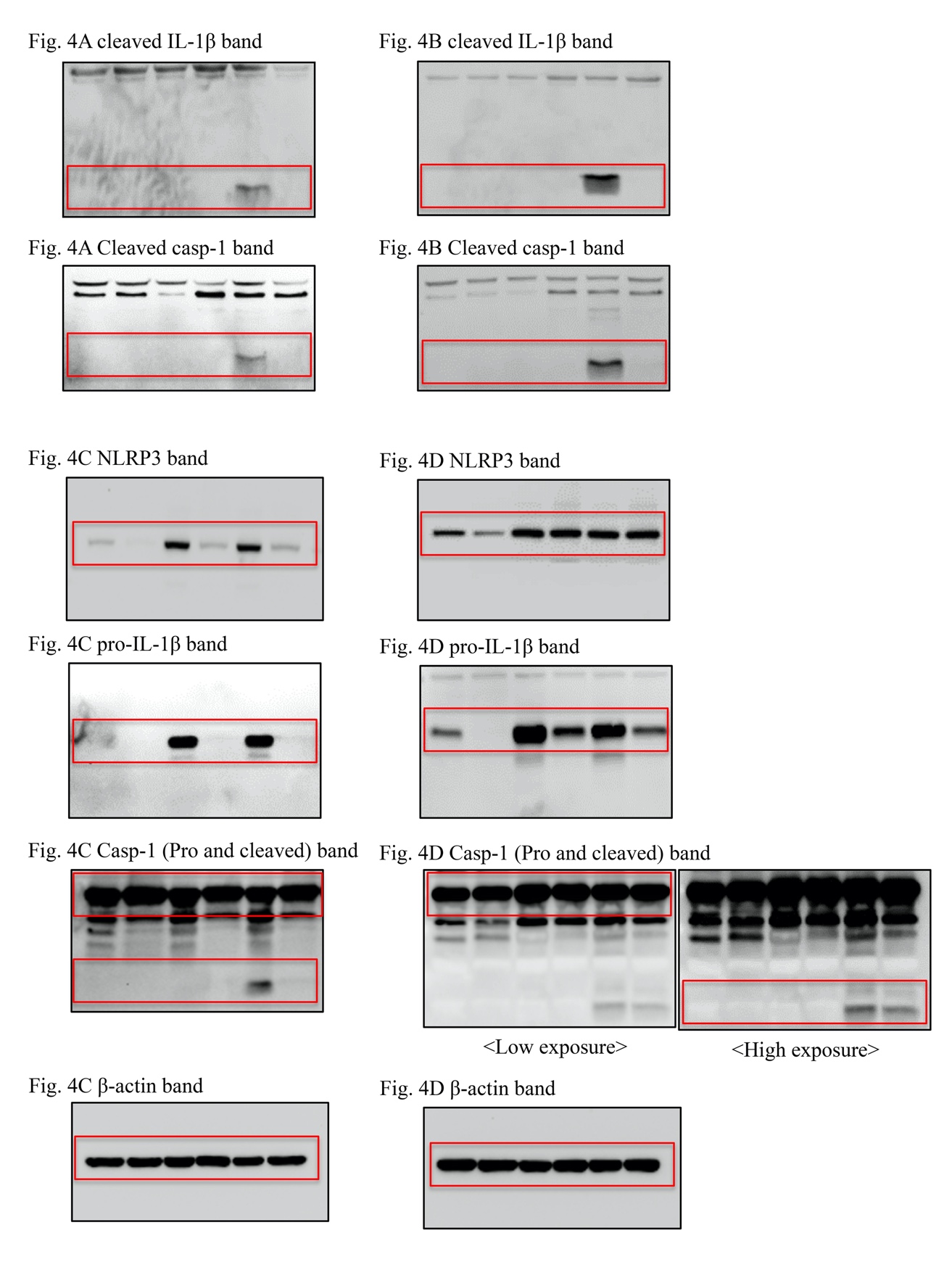


**Figure S1. The original blots of cropped images shown in the main figures 4.**

**
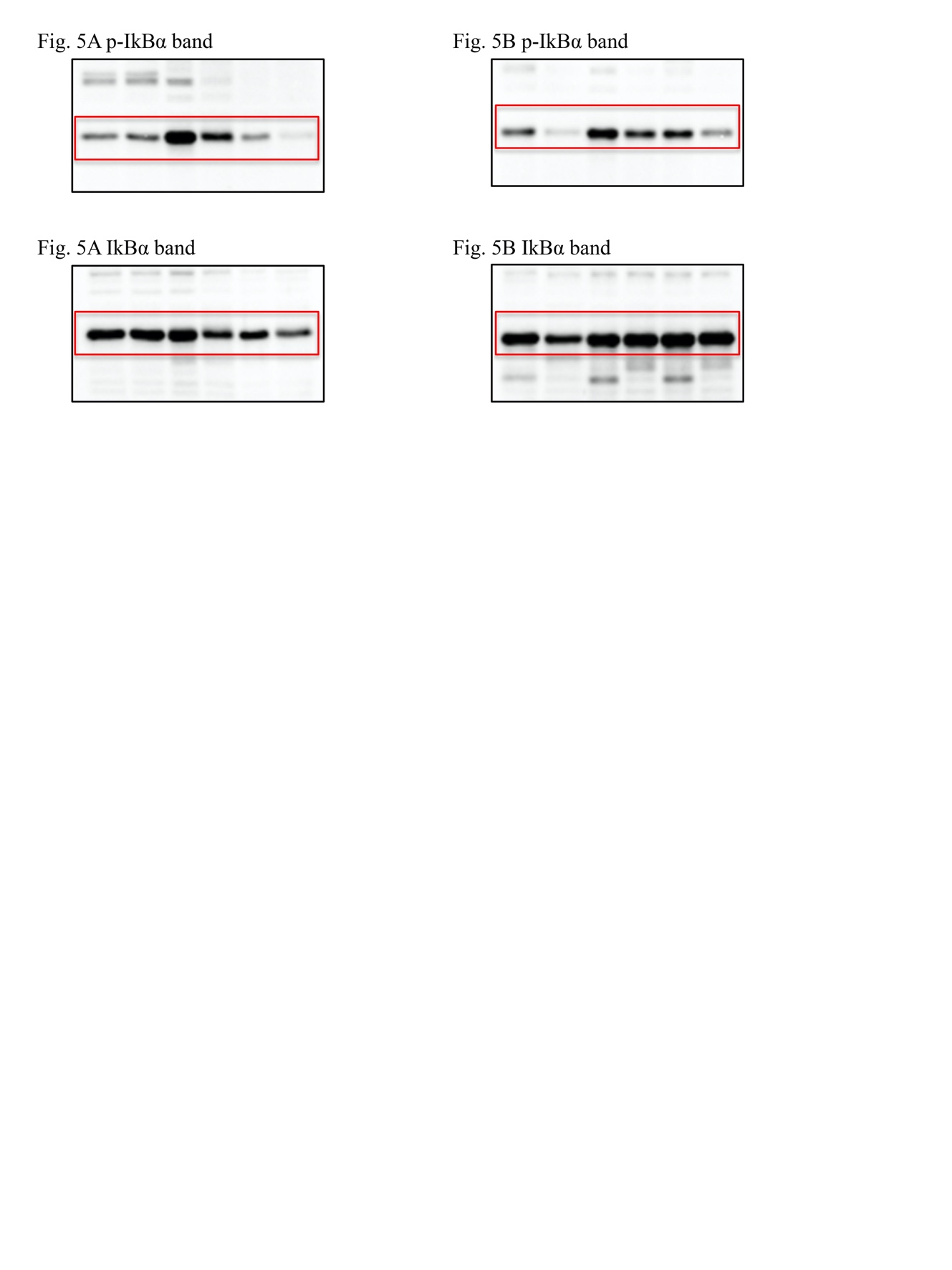
**

**Figure S2. The original blots of cropped images shown in the main figures 5.**

**
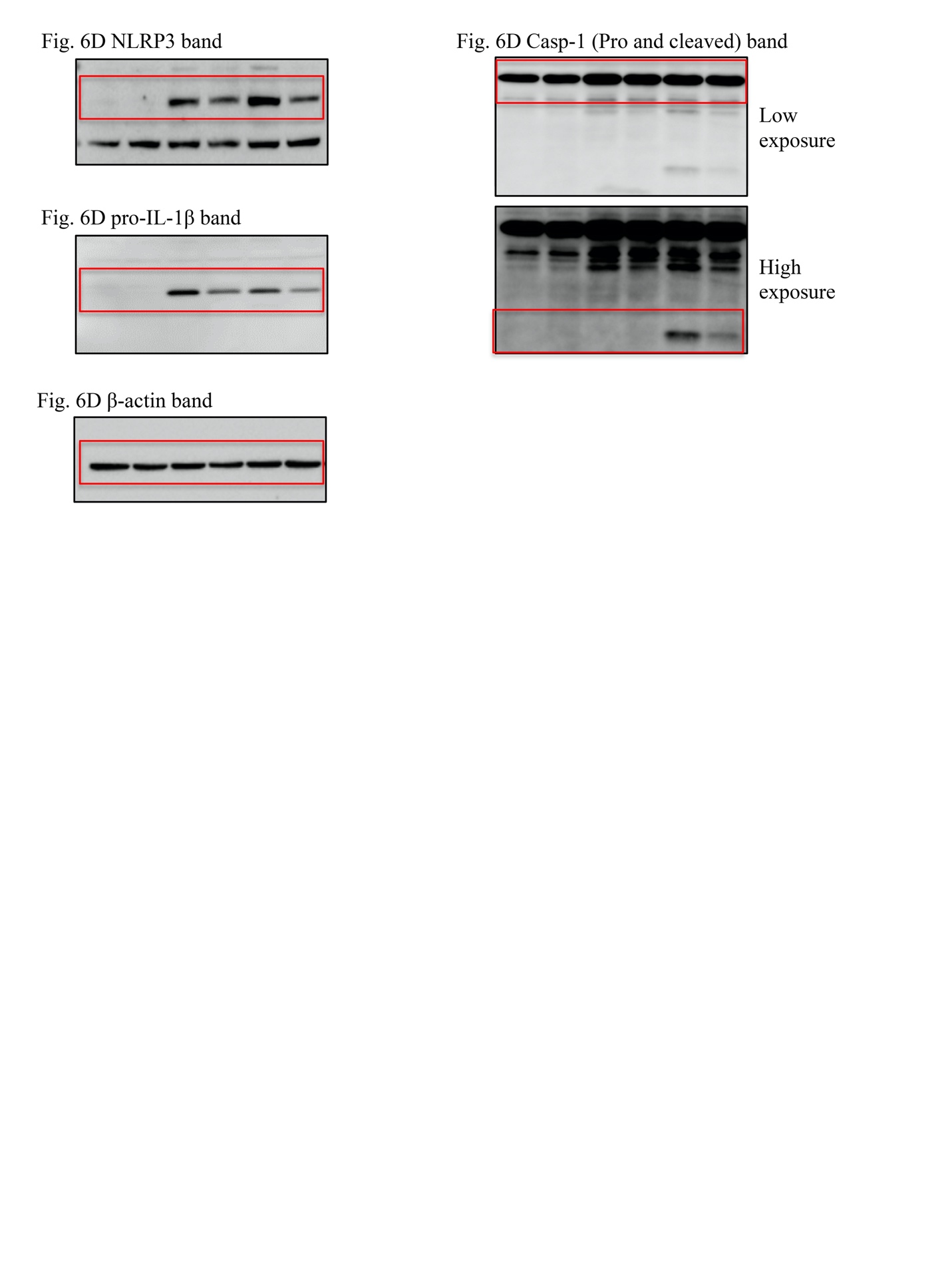
**

**Figure S3. The original blots of cropped images shown in the main figures 6.**
